# On Deflationary Accounts of Human Action Understanding

**DOI:** 10.1007/s13164-018-0386-3

**Published:** 2018-02-17

**Authors:** Emma Borg

**Affiliations:** 1 Reading Centre for Cognition Research, Department of Philosophy, University of Reading, Reading, RG6 6AA UK; 2 Australian Research Council Centre of Excellence in Cognition and its Disorders, Macquarie University, Sydney, Australia

## Abstract

A common deflationary tendency has emerged recently in both philosophical accounts and comparative animal studies concerned with how subjects understand the actions of others. The suggestion emerging from both arenas is that the default mechanism for understanding action involves only a sensitivity to the observable, behavioural (non-mental) features of a situation. This kind of ‘smart behaviour reading’ thus suggests that, typically, predicting or explaining the behaviour of conspecifics does not require seeing the other through the lens of mental state attribution. This paper aims to explore and assess this deflationary move. In §1 I clarify what might be involved in a smart behaviour reading account via looking at some concrete examples. Then in §2 I critically assess the deflationary move, arguing that, at least in the human case, it would in fact be a mistake to assume that our default method of action understanding proceeds without appeal to mental state attribution. Finally in §3 I consider briefly how the positive view proposed here relates to discussions about standard two-system models of cognition.

When philosophers consider the so-called ‘problem of other minds’, there are a cluster of concerns they might be interested in, including:

i. How do I know you have a mind like mine?
ii. How do I know what subjective states you enjoy?
iii. How do I predict and explain your behaviour?

(i) is the classical Cartesian worry – the sceptical concern that nothing in the available evidence rules out the possibility that I am surrounded by complex automata, that I alone am minded. (ii) reflects concerns pressed by Nagel and Shoemaker, amongst others, that the qualitative, phenomenal states had by others might be radically different from mine and indeed might be constitutionally unknowable for me. (iii), on the other hand is a much more practical kind of worry – it asks how we manage to interact with others on a day-to-day basis – and as such it has been a focus of investigation not only in philosophy but also in psychology and related disciplines. In philosophy, a focus on (iii) led to the development of theory-theory vs. simulation accounts of mindreading and, in psychology, to related views of Autistic Spectrum Disorder as involving a kind of ‘mindblindness’ (see Baron-Cohen 1995). Recently, however, a different and, I want to suggest, more deflationary, answer to (iii) has emerged across a number of fields, whereby, at least in typical cases, behaviour prediction and explanation requires no access to other minds at all since it rests on what we might call ‘smart behaviour reading’. On this model, we come to understand and predict the behaviour of others not via seeing them through the lens of mental state attribution but more directly, through a sensitivity to their physical context and bodily motions.

To explore this proposal I will first (§1) clarify what might be involved in adopting a smart behaviour reading account, in part by looking at some concrete examples, and I will suggest that advocates of smart behaviour reading are right to stress the role of behaviour and environmental conditions, together with an important role for associationist learning, in this area. However I will go on to argue (§2) that genuinely deflationary versions of the view face serious problems, since (at least in the human case) it is only against a background of mental state attribution that behaviour reading can actually succeed in making actions predictable and explicable. Finally (§3) I conclude by suggesting (contra some in this area) that the view proposed here – whereby *both* mental state attribution and behaviour reading typically have a role to play in human action understanding – should not be construed as an instance of the now classic two-system view of cognition (proposed by Kahneman and others).

## Mental State Attribution vs. Smart Behaviour-Reading

Once upon a time in philosophy of mind if one talked of different accounts of how we understand one another’s behaviour, the accounts in question would have been theory-theory and simulation theory approaches.^1^ While the philosophical debate between theory-theory versus simulation-based accounts did not, I take it, get decisively settled, at least some theorists came to the conclusion that a complete account of how we understand others would probably end up involving both approaches (see, e.g., Goldman 2006). This idea – that our ability to understand others, and thus create and access a social world, might be underpinned not by a single, homogenous mindreading mechanism, but instead by multiple, distinct processes – is, to some extent I think, echoed in recent ‘pluralist’ moves which posit multiple distinct strategies for human action understanding. For instance, Andrews 2012 and Fiebich and Coltheart 2015 argue that there are (at least) two different ways in which subjects come to understand the actions of others:

i) Mental state attribution: A witnesses B’s behavioural and environmental cues and uses these to ascertain B’s mental state. A uses these attributed mental states to predict and explain B’s behaviour. E.g.:

- Jill is wearing running clothes and appears breathless and red in the face. She is filling a glass from the tap. A infers: Jill is thirsty and wants a drink. A predicts: Jill will drink from the glass she is filling.

ii) Smart behaviour-reading: A witnesses B’s behavioural and environmental cues and predicts and explains B’s behaviour on the basis of these cues. E.g.

- Jill is wearing running clothes and appears breathless and red in the face. She is filling a glass from the tap. This behaviour in this context makes it evident to A that Jill will drink from the glass she is filling.

(i) captures a kind of standard folk psychological approach to action prediction and explanation, maintaining that we come to understand another’s actions by seeing the agent through the lens of mental state attribution; we ‘mentalise’ the subject and take the mental states posited to underpin the action in question. (i) is quiet on whether we engage in this mental state attribution via an explicit bit of reasoning utilising a subject’s theory of mind or whether we come to consider the mental states of others via the kind of imaginative exercise posited by (at least explicit versions of) simulation theory, but however we do it, (i) claims that (at least some) action prediction involves viewing a target as in possession of mental states. It is far from clear, however, whether or not (ii) also makes this commitment. To decide this, we need to know more about what is involved in a behaviour reading account. Thus in §§1.i-1.iii I’ll sketch three possible versions of a behaviour reading account, two of which I’ll claim clearly do provide deflationary accounts of human action understanding and one (Heyes’s ‘submentalizing’ approach) which may not.

One important point to note initially, however, is that smart behaviour-reading, in whatever form we adopt, is *not* supposed to be equivalent to classic Behaviourism. This is for at least two reasons: first, there is no commitment to the idea that mental states *reduce* to stimuli-response pairs, rather the claim is simply that action prediction and explanation can proceed directly on the basis of sensitivity to the behaviour of others. In this sense it is an epistemic claim about how we come to know what someone else will do, not a metaphysical theory about what mental states are. Second an advocate of smart behaviour-reading, but not an advocate of Classic Behaviourism, can accord a wealth of rich cognitive capacities to subjects: they can allow that subjects entertain genuine mental representations which generalise across distinct stimuli, undertake inferences, track associative relations, and so on (the *smart* element of smart behaviour-reading). Thus we should be clear that, on the smart behaviour reading model, subjects are genuine ‘cognitive creatures’ (Penn and Povinelli 2013: 75).

### Embodied Versions of Smart Behaviour-Reading^2^

Embodied accounts of action understanding are part of a general move away from traditional, Cartesian accounts within philosophy of mind. They hold that our understanding of others is primarily subserved by shared motor competencies which allow us to predict and explain behaviour directly via sensitivity to situated action. The thought here is not so much that ‘we see the mental states of others in their behaviour’ (as in Wittgenstein’s account of mental states like pain, see McDowell 1978: 304) as that we normally have no need to consider the mental states of another, as abstract representational states, at all, for engaging in an appropriate motor resonance with that other turns out to be sufficient for action understanding.^3^ As Spaulding 2012: 433 writes:

[A]ll ESC [Embodied Social Cognition] accounts hold that our capacity for social cognition is not based on ascribing mental states to others. Rather, what underlies our ability to understand and interact with others is the capacity for more basic, non-mentalistic, interactive embodied practices.

Many embodied, motor-resonance accounts distinguish between primary intersubjectivity and secondary intersubjectivity, where the first developmental stage involves skills such as the detection of intentional action and the recognition of emotion from bodily states, while the latter involves the development of joint attention, such as communicating about objects in the shared environment (Gallagher and Hutto 2007). Yet neither stage is held to involve representation or attribution of abstract mental states. As Hutto 2008: 51 claims, “Our primary worldly engagements are nonrepresentational and do not take the form of intellectual activity”.^4^

Embodied approaches to action understanding are sometimes fleshed out in terms of an appeal to the ‘mirror neuron system’.^5^ Mirror neurons are neurons which fire in two distinct conditions: when a subject δ’s and when a subject witnesses a conspecific δ-ing (see, e.g., Gallese 2001, Rizzolatti and Craighero 2004, for discussion.). So, for instance, it turns out that certain motor regions of my brain will be in the same state when I reach out to grasp a glass of water and when I see you reaching out in the same way to grasp a similar object. Many theorists have suggested that this kind of neural mirroring within motor systems underpins action understanding: I understand that you are grasping the glass in order to drink because your action triggers a neural state appropriate to grasp-to-drink actions in my brain (see, e.g., Sinigaglia 2008, Gallagher 2009, Craighero 2014; for sceptical perspectives see, e.g., Borg 2007, Hickok 2008). Yet whether we choose to spell out an embodied approach in terms of the mirror neuron system or in some other way, it seems very likely that the cognitive mechanism in question here will be one of associationist learning (see, e.g. Heyes 2012): what triggers my prediction that your grasp of the cup will be followed by your moving the cup towards your mouth is the fact that, in relevantly similar situations, I’ve most often witnessed that kind of gesture – grasping of cup – being followed by that kind of movement – cup towards mouth.^6^ What behaviour-tracking models do, it seems, is allow for the prediction (and perhaps the explanation) of action via learnt statistical regularities of behaviour (for this kind of account of action understanding, then, it seems plausible to say that we predict what we have lived).^7^

Embodied approaches to action understanding do, then, obviate the appeal to mental state attribution – we predict the actions of others without recourse to reasoning about abstract mental states – and thus the approach qualifies as deflationary in the sense of this paper. The claim is that standardly we understand and predict the actions of others not via some form of mindreading (mental state attribution) but via a (most probably learnt) sensitivity to common behavioural patterns. For the most part we co-ordinate our actions with those of others not because we view the other through the lens of abstract symbolic states like belief and desire but rather because we have come to associate a certain kind of behaviour in a certain kind of setting with a certain kind of outcome.

### Comparative Versions of Smart Behaviour Reading

Premack and Woodruff first explicitly posed the question ‘does the chimpanzee have a theory of mind?’ in their classic eponymous 1978 article and a positive, though qualified, answer to their question has recently been suggested by, amongst others, Call and Tomassello 2008: 191 (revising the view of Tomasello and Call 1997):

In a broad construal of the phrase ‘theory of mind’…the answer to Premack and Woodruff’s pregnant question of 30 years ago is a definite yes, chimpanzees do have a theory of mind. But chimpanzees probably do not understand others in terms of a fully human-like belief–desire psychology in which they appreciate that others have mental representations of the world that drive their actions even when those do not correspond to reality. And so in a more narrow definition of theory of mind as an understanding of false beliefs, the answer to Premack and Woodruff’s question might be no, they do not.

The experimental work which prompts Call and Tomasello’s qualified ‘yes’ here involves naturalistic tasks (often involving competitive food scenarios), where chimpanzees’ behaviour apparently demonstrates a grip on the mental states of conspecifics. So, for instance, work by Hare et al. 2001 shows that, if a subordinate chimp X witnesses food being hidden in a shared environment, then X will later preferentially approach that food if they are aware that a dominant chimp Y did not see it being hidden (e.g. because Y’s view was occluded). This seems to show that, at least in some settings, chimpanzees are able to reason about what another subject can or can’t see, and this constitutes, it is argued, at least some capacity for mental state attribution.

However, in a range of works, Povinelli and colleagues have objected to this conclusion, arguing that “comparative researchers have consistently failed to specify what *unique causal work* is being performed by nonhuman subjects’ [Theory of Mind] system that could not have been performed by a sophisticated cognitive system representing and reasoning about observable behaviours alone” (Penn and Povinelli 2013: 76).^8^ For instance, as they point out, a mindreading explanation isn’t *demanded* by experimental evidence like that given above. For although the animals *could* be reasoning about what a conspecific can see (mental), they could also be basing their behaviour on simple observable facts such as whether or not it is possible to draw an uninterrupted line from the target’s open eyes to the food source while it is being hidden (line of gaze, non-mental). Thus, as Lurz et al. 2014: 428 put it, on this model:

[A]nimals predict the behaviour of others by means of cognitive processes that range over nonmentalisitic representations of behavioural and environmental cues and relations. Some of these cues and relations can be rather specific…such as ‘torso facing forward’ or ‘hair bristling’. Others can be more abstract…such as ‘threat display’, ‘orienting towards an object’ or ‘manipulating an object in the most efficient way within the constraints of the setting’. What makes these representations of such behavioural and environmental cues nonmentalistic is that the animal can represent them as such without having any understanding of the mental states that may be causing or associated with them in other agents or themselves.

It seems that we can (somewhat roughly) formalise the argument here, which is sometimes labelled as ‘Povinelli’s Problem’, as follows:

1. An inference by A to assign a mental state to B must take as its evidential base only observable facts about B’s behaviour and environmental cues.^9^
2. Given this evidential base, an alternative explanation of A’s performance is available that appeals entirely to behavioural and environmental features.
3. Do not treat others as reasoning about mental states if a lower level explanation is possible (parsimony).^10^
4. Treat non-human animals as smart behaviour readers rather than as mental state attributers.

Povinelli’s Problem has its main home in the context of debates about primate mindreading. However, the structure of the argument means that it could, at least in principle, extend more widely. For instance, the Problem straightforwardly extends to attributing reasoning about mental states to pre-verbal infants (see Baird & Baldwin 2001, Gergely and Csibra 2003, Perner and Raffman 2005). Since pre-verbal infants are not in a position to tell us that they are engaging in mental state attributions, and since we can, *ex hypothesi*, explain all of their predictive success without positing mental state attribution, perhaps we should refrain from treating them as reasoning about unobservable mental states at all and instead accord them the more concrete skill of smart behaviour-reading. Furthermore (although we should be very clear that this is not Povinelli’s position) it seems that we *could* also consider extending the range of the argument to cover any case of putative mental state attribution where we lack verbal testimony that the subject really is exploiting knowledge of mental states.^11^ That is to say, any time a subject doesn’t provide explicit (e.g. verbal) evidence to tell us they are engaging in mental state attribution, an application of the above argument would tell us to analyse them as engaging in the less demanding practice of behaviour reading instead.^12^ In this way, Povinelli’s Problem would apply to all ‘unreflective’ instances of adult social cognition (see Hurley & Nudds 2006, Butterfill & Apperly 2013): although in the case of adult humans we have verbal testimony to support the claim that they have the *capacity* to engage in mental state attribution, an extended application of Povinelli’s Problem would prompt us not to posit the *exercise* of this capacity unnecessarily. However widely we choose to extend the argument, though, it is clear that the resulting view of action understanding is deflationary in the sense outlined above: it tells us that, for any creature where we decide to apply Povinelli’s Problem, the result will be a deflationary account of action understanding in that creature – we will model the way in which the creature predicts (and perhaps explains) the actions of others without any recourse by it to reasoning about unobservable mental states.

### The Submentalizing Approach

Finally I want to sketch a third, superficially similar approach, which we might also think constitutes a smart behaviour reading account of action understanding, namely the submentalizing approach championed by Cecilia Heyes (e.g. Heyes 2014), and suggest that in fact it falls beyond the concerns of this paper. Heyes wants to challenge the experimental evidence presented in support of the existence of implicit mentalizing (i.e. the tacit consideration of the mental states of others) by showing that non-mentalizing explanations of the behaviour in question (what Heyes’ calls ‘submentalizing explanations’) can in fact be given. These explanations appeal to entirely domain general processes, i.e. processes that can come into play regardless of whether the stimulus is social or asocial.

So, for instance, consider work by Samson et al. 2010 where a subject’s ability to assess the number of dots in a subsequently presented scene was found to be impaired when that scene involved another person (or avatar) and where only a subset of the dots visible to the participant were visible to the avatar. Thus, if the display showed a room containing an avatar facing the left-hand wall and the dots in the room were arranged with two showing on the left-hand wall (so ‘seen’ by the avatar) and one showing on the right-hand wall (so ‘unseen’ by the avatar), participants were slower in judging that the number of dots in the room was three (i.e. slower than they were when shown a similar scene minus the avatar). The suggestion is then that this slowing in performance is the result of an interference effect from unconscious reasoning about the number of dots visible to the avatar. That is to say, although nothing in the experimental paradigm requires the participants to reason about what the other can see, participants cannot help automatically considering the other person’s perspective. This seems to show that implicit mentalizing occurs fast and without conscious control. Furthermore, other experimental evidence (such as preferential looking in false belief tasks) suggest that this skill emerges early in typically developing infants. Implicit mentalizing thus looks like a good candidate for explanation via a nativist, modular cognitive capacity.

However, considering this and other experimental work, Heyes suggests that the conclusion of implicit mentalizing is too swift, since alternative submentalizing explanations are possible. So, in an extension of Samson et al.’s paradigm, Heyes found that the same results could be obtained from a stimulus which replaced the avatar with a directional arrow: subjects were slower at responding that there were three dots in a room if two of the dots were located in front of an arrow pointing in that direction and one was not. What this seems to show (given the plausible assumption that subjects are not assigning faux mental states to the arrow) is that the interference effect comes simply from making some sub-set of the dots more salient to participants (so, as Heyes 2014: 134 puts it, it is the directional features of the avatar that are important, not its agentive features). An explanation of the delay can thus be given in terms of entirely domain general principles concerning salience manipulation.

Heyes’s submentalizing approach seems closely aligned with the above accounts of smart behaviour-reading, since all three accounts are designed to show that cases which prima facie might be thought to require mental state attribution can in fact be explained in non-mentalized terms. Thus we might think to incorporate Heyes’s account alongside these others. However, I think there may be reasons to resist such a move (at least from the perspective of this paper). First, the focus of smart behaviour reading accounts (as construed here) is on challenging the role of mindreading in predicting the actions of others, while Heyes is interested in challenging a much wider claim, namely that implicit mindreading is responsible for a range of effects witnessed in self-initiated behaviour. So, for instance, in the dot experiment, participants are not trying to predict the movements of the avatar – they are not engaging in action prediction at all – rather they are trying to perform their own act of calculation (with putative interference from a mindreading task). A submentalizing explanation of these kinds of cases then might leave untouched the cases we are really interested in here. That is to say, the possibility that implicit mindreading isn’t involved in something like the dot experiment still leaves open the possibility that it is involved when we are actively engaged in predicting the actions of others.^13^ This relates to the above noted domain-generality of submentalizing processes, which are not selective in terms of input and thus may be operative whether or not a stimuli is social. This is very unlike behaviour-reading accounts proper, which are highly domain-specific, coming into play only when a stimuli is perceived by an agent as social.^14^ Finally, we should note that one of the main thrusts of Heyes’s work is to challenge moves towards nativism by showing how associationist learning could be responsible for the behaviour in question. However, as I will suggest below (§3), claiming that mindreading is involved in behaviour prediction and explanation doesn’t necessarily entail making any specific claims about the status of the cognitive processes underpinning mindreading (i.e. as necessarily modular or innate). So the approach to be advocated in this paper might be thought to cross-cut Heyes’s concerns in ways that make it problematic to treat her submentalizing approach as a direct target. Thus in what follows I will focus on the sort of deflationary approach to action understanding provided by embodied accounts (§1.i) and emerging out of comparative animal research (§1.ii).

### The Role of Smart Behaviour Reading in Action Understanding

Advocates of smart behaviour reading approaches maintain that traditional mentalising approaches vastly overestimate the prevalence of mental state attribution. For instance, consider cases of simple co-ordination (e.g. avoiding people in corridors, or passing an object to someone) or contexts which are highly stereotyped (driving, purchasing items, etc.) – here they argue that we could rely on purely non-mentalistic mechanisms, simply associating behavioural cues with outcomes (Fiebich and Coltheart 2015: 240 call these kinds of cases ‘behaviour expectations’).^15^ As Lurz et al. 2014: 446 write:

It is quite obvious that when predicting an opponent’s behaviour in a sporting match, humans use a quick and effortless behaviour reading mechanism, and that in highly stereotypical social interactions (e.g. ordering meals at restaurants, or buying food at a butcher shop) humans employ behavioural rules that range over representations of social roles and deontic rules…Also there is growing empirical evidence that the performance of children (and adults) on social competence tasks is sometimes controlled by behaviour-rule mechanisms rather than mindreading mechanisms.^16^

We have a picture then of the default mechanism for understanding the actions of others as resting not on some complex, inferential mental state attribution and an application of general (perhaps innate) folk psychological rules, but rather as a direct, unmediated (and probably learnt) response to the situated bodily behaviour of another. So should we agree with this kind of deflationary move, allowing that the standard mechanism for action prediction involves behaviour reading and not mindreading? In the next section I want to argue that, at least with respect to the understanding of human action, we should not. For even though I think some forms of action understanding do (perhaps contra older views in the philosophy of mind) lean heavily on behavioural contingencies and learnt associations, in general I want to suggest that we cannot hope to understand human action without a rich background of mental state attribution.

## Problems for Behaviour Reading Accounts of Human Action Understanding

Contra deflationary views, I think that our basis for understanding the actions of others cannot be construed as purely behavioural. At heart, this is because individuating the behaviour and situations we are interested in, and determining which of potentially many behavioural rules to apply, in itself depends on a background of mental state attribution. Perhaps in the case of non-human primates it could turn out that purely physical descriptions of behaviour could suffice (though in fact I think this is unlikely), but to fix or apply the kind of rich behavioural rules that we need to predict the behaviour of other humans, we cannot avoid attributing mental states to those others. To see this I want to rehearse four objections, all of which (in somewhat different ways) challenge the idea that human action understanding could be a matter of smart behaviour reading alone.

### Creation and Application of Behavioural Rules Presupposes Mental State Attribution

Smart behaviour reading approaches neglect, I suggest, the role mental state attribution plays both in individuating learning situations and in determining which behavioural rules to apply post-learning. To see this, imagine that, during a learning phase (when we are constructing our behavioural rules via a process of associationist learning) we come across a target B who does not eat available food in an ordinary ‘eating’ context (i.e. where a subject who hasn’t eaten for some time is presented with available food). Is this evidence *against* the behavioural rule that ‘a subject in an ‘eating’ context will typically start to eat presented food’? Or, alternatively, is it evidence *for* a rule such as ‘a subject in an ‘eating’ context when presented with food s/he doesn’t like will typically not eat that food’? To decide this, and thus to learn the associationist lesson the situation teaches us, it seems we need to consider the mental states of the other (i.e. whether or not they like the food presented). Furthermore the association-based behavioural rules we acquire are themselves extremely likely to make reference to the mental states of targets. For instance, a simple behavioural rule such as ‘a person who sees an animal x as a threat will avoid x’ has the perspective of the other built in to it – to predict avoidance behaviour it doesn’t matter whether x is or is not a genuine threat, what matters is whether the target sees x as a threat – and this is just to consider the cognitive states of the other. Although it is surely right that habituation to actions (in context) leads to anticipation of the habituated outcome when the subject is later exposed to the same kind of action in contexts which sufficiently resemble those encountered in the learning phase the problem is that, first, carving up the learning phase actions in a way that will yield statistically reliable regularities will often involve conceiving of the target under mental state descriptions (i.e. does the target like the food? Do they recognise the seen shape as a predator? Do they view another conspecific as rewarding or not?) and, on the other, deciding that a current scenario is enough like one encountered during a learning phase will itself involve judging whether the mental framework ascribed to the target is sufficiently like the one ascribed to the agent encountered in the learning phase.

The kind of challenge envisaged here should, I think, seem familiar, for it echoes a standard objection to Classic Behaviourism, namely that there is no individuating typical stimuli and response pairs for human action without consideration of the mental domain.^17^ Of course, the objection doesn’t carry over absolutely directly, for recall that there is no aim of eliminating mental states in favour of stimuli-response pairs on a smart behaviour reading view. Yet still I suggest it poses a problem for smart behaviour-reading: if the claim of this approach is that we can understand and predict the actions of others without any recourse to mental state attribution, then it must be that we can characterise the evidence base for our understanding in entirely non-mental terms (this was premise 1 of Povinelli’s Problem above). Yet on reflection this seems like a mistake: to predict your behaviour it matters how I think you conceive of or represent the current situation.^18^ Does your selection of that dish provide me with evidence that you like ice cream? Well, only if I think that you see it as ice cream. If you eat it does that provide me with evidence that you’ll eat it again next time? Well, not if I think you only ate it this time to be polite. Statistical or associationist learning is hugely useful for us but it would be a mistake to think that, with respect to the behaviour of others, we can characterise the learning situations themselves or how we later decide which learnt rules to apply without some sort of appeal to the mental realm.^19^

This is not to say that every token act of action prediction must involve an explicit mental state attribution to another. I think it is extremely plausible that, for instance, in a game of tennis I come to predict whether an opponent is going to serve out wide rather than down the line via sensitivity to extremely subtle, probably sub-consciously registered physical ‘tells’ on their behalf. Sensitivity to the delicate web of purely physically described behaviour that the other displays clearly matters. But even in cases like these, it seems the behavioural cues can only trigger my action prediction within a characterisation of the situation that takes into account the perspective of the other: it matters, for instance, that I view my opponent as intending to engage in a game of tennis with me rather than intending to try and hit me with the tennis ball, etc.^20^ A purely physical description of the subject and her environment, stripped of any kind of appeal to the intentional and affective states of the other, would be, I claim, simply inadequate for characterising the situations we encounter in ways that would either enable statistical learning about behaviour to take place or identify which of a plethora of potentially applicable behavioural rules one should actually apply on a given occasion.

### Character- and Stereotype-Based Predictions

Models of action understanding which rely on tracking observable regularities of behaviour run the risk of breaking down in situations where it is the *individual* which is important and where the important characterisations of the individual are not exhausted by behaviour. So consider the following examples:

- Jill is in a maths class which has just been asked a simple question about a calculation on the board and I’m considering whether or not Jill will raise her hand to answer the question. Past experience tells me that, in mixed maths classes, girls are less likely than boys to raise their hands, so I could predict that she won’t raise her hand. But I believe that Jill is good at maths and also that she is concerned about female representation in STEM subjects. On this basis (although never having seen her in class) I predict that she will raise her hand.
- Karthik is wearing gym clothes, is sweating and is filling a glass with water. I recall that last night he and I watched a TV programme which claimed that sugared water was the best fluid to drink after strenuous exercise. Thus I predict that he will not drink from the glass he is filling but will instead carry it to where the sugar is kept.

Obviously, these are just toy examples of genuine action prediction but what they help draw out, I think, is that individualistic behaviour prediction often doesn’t take place in a cognitive vacuum. Much action prediction depends on what we know about a target’s background beliefs and desires, personality and character traits, likes and dislikes, and the power relations and social mores in play in a given exchange.^21^ Furthermore, it seems that appeals to character traits, etc., do not simply reduce to claims about behaviour (as a fully deflationary advocate of behaviour reading might seek to maintain). For, first, character traits allow us to make predictions in novel situations. I may never have seen Jill in class before but knowledge of her character allows me to make a prediction about what she will do: I may have witnessed Jill being brave in other situations, so predict that she will behave bravely in this one. Yet without viewing her in character-based, mentalized terms (i.e. as brave), I would have no way to group those past situations alongside this one. Secondly, it seems that we also use traits as a useful tool in action understanding but where ascription of those traits is independent of a target’s behaviour. For instance, work in social psychology reveals that we often assign individuals to stereotype groups on the basis of crude surface features and use these stereotypes to guide subsequent predictions of behavior, notably even if the individual whose behavior is being predicted regularly fails to perform in line with the assigned stereotype. So if a subject, A, is someone for whom gender stereotypes are particularly salient, they may predict that a female target B will behave in a stereotypically feminine way, even if A has in fact witnessed B regularly failing to act according to gender stereotype in the past (Andrews 2012).^22^ It seems then that our predictive and explanatory practices involving character-traits and stereotypes reveal points at which action prediction relies on something other than witnessed behavioural regularities, looking instead to the kind of mental characteristics a target is held to possess.

### Linguistic Communication

As Andrews 2012 notes, smart behaviour reading accounts will need to appeal to a heuristic involving linguistic cues. For instance, they might posit a simple rule such as:

SAY: Targets tend to do what they say they will do.

However, it should be obvious that this heuristic will qualify for use within a non-mentalistic account of social cognition *only* if it is possible for a subject to recover what a target says they will do – the content of their communicative speech act – without considering the mental states of the target. Yet, at least according to one highly influential model of communication, this is not the case. Anyone who accepts a broadly Gricean account of the nature of communicated content will hold that (on some or all occasions) it is not possible to recover ‘what is said’ by a speaker without thinking about what that speaker intends to convey.^23^ Imagine, for instance, the speaker who says, when passing a bakery, ‘Can we stop? I’m hungry’. In the right context, the speaker is likely to successfully imply that they want to buy something in the bakery, although they certainly don’t directly assert this. Grasping the implicature will matter for predicting what the target is likely to do next (go into the shop, say), but working out what someone implies, on almost all accounts, depends on consideration of the intentional states of the speaker: important elements of linguistic evidence then seem to depend on reasoning about what a speaker intended to convey. Thus a simple rule like SAY seems to gloss over a background appeal to mental states which is required in order to determine what it is that a speaker has actually said (in the sense of what she has communicated). Once again then the worry is that, although advocates of smart behaviour reading can give the superficial appearance of obviating an appeal to mental states, when we push a little harder on how we characterise or arrive at the behaviour we are interested in, what is revealed is a background dependence on mental state attributions.

### Simplicity and Parsimony

As noted in §1.ii, a core element of an argument in favour of behaviour reading accounts involves an appeal to a simplicity or parsimony claim such as: explain data in terms of the simplest possible cognitive endowment for a subject. However, as Elliott Sober has argued at length (see, e.g. Sober 2009), although such a claim seems intuitively to speak in favour of explaining action understanding (in particular amongst non-human subjects) in terms of behaviour reading only, this intuitive appeal may not actually be born out once we turn to consider the mechanics of theory selection in any detail. For instance, understood as a claim about probability, the appeal to parsimony here amounts to the claim that it is less likely that an animal possesses first and second order intentionality (i.e. has mental states *and* can reason about mental states) than that it has first order intentionality *only* (i.e. has mental states but does not reason about mental states). Yet any such claim about distribution of probability needs justification and Sober argues that this necessary justification is missing (it cannot, for instance, be supplied by an appeal to Darwinian evolution).^24^

Furthermore, as many theorists have pointed out, the claim that behaviour reading accounts have greater simplicity than mentalising approaches is much more controversial than might initially be thought. For reasoning about mental states allows rules to be stated at a level of generality not available to behaviour reading accounts. As Call and Tomassello 2008: 187 note with respect to the animal literature: “[Povinelli and colleagues] cling to the hypothesis that chimpanzees understand only surface-level behavior (forming ‘behavioral rules’), and indeed this explanation is almost always possible for any single experiment. But there are now in many cases multiple experimental paradigms all aimed at a single psychological state – each presenting chimpanzees with a highly novel problem – that makes the positing of learned behavioral rules a difficult explanatory strategy.” For while a purely behavioural approach will need one rule for each discrete piece of behaviour (reaching for an apple in this manner or that manner, reaching for an apple in the presence of this environmental cue or that one, etc.), a mentalising approach can state things in ways that generalise across a range of possible behavioural realisations (i.e. stating things simply in terms of conditional claims such as ‘if A wants p and believes doing q is a way to get p, then ceteris paribus A will do q’). Thus, if simplicity is a matter of ‘counting rules’, it seems highly likely that mentalising approaches will in fact turn out to be simpler than behavioural ones.

What (2.i)-(2.iv) show, I think, is that we have good reason to resist a genuinely deflationary approach to action understanding (at least in the human case): we cannot, in general, explain our ability to predict and explain the actions of others on the basis of an appeal to physically described behaviour and environmental features alone. For, first, learning the crucial behavioural regularities themselves, and then determining whether or not a current situation is an instance of a learnt regularity, requires consideration of mental states (how the target conceptualises the situation, what mental states they bring to the context). Second, behaviour-prediction in terms of character-traits or stereotypes goes beyond merely observed behavioural regularities, since we are sometimes willing to take a target to possess a given trait or character even if we have not witnessed it being expressed in the majority of contexts. Third, we have good reason to think linguistic communication involves (constitutively or in large part) reasoning about speaker intentions, thus to allow verbal behaviour to constitute part of our evidential base in action prediction, the system will need to incorporate background appeal to intentional states. Fourth, claims of simplicity speak as much, if not more, in favour of mentalised models as behaviour reading ones. Thus, while recognising the crucial role behaviour-tracking plays in action understanding, I want to suggest that a move to eradicate mental state attribution in favour of behaviour-tracking as the default method for human action understanding is not, ultimately, feasible.

## Is Action Understanding an Example of a Classic Two-System Skill?

In closing, I want briefly to consider (and reject) one possible reason why a deflationary move in this area might have been thought attractive in the first place. For alongside a general suspicion about symbolic representation of abstract, unobserved mental states, I think moves towards smart behaviour reading have also been motivated by a recognition of the speed and automaticity of many of our judgements in this area (the fact that action understanding often looks very much like a classic ‘System 1’ operation; see, e.g. Kahneman 2011), together with a worry that a full-blown mindreading system could not capture these features.^25^ However, I want to suggest that it would be a mistake to think that, by its nature, mental state attribution must be slow, effortful and under conscious control. First, as adults, although we sometimes reason deliberatively about the minds of others, on very many other occasions we proceed to quick, automatic judgements of mental states (her grimace leads me to think that Jill doesn’t like beetroot, his sweating leads me believe that Jack feels hot) which require no slow, conscious reasoning. Second, the evidence of very early onset sensitivity to the mental states of others (as displayed in preferential looking versions of false belief tasks and as required for language acquisition) speaks in favour of a more automatic, non-reflective kind of access to the mental states of others. Third (as Apperly and Butterfill 2009: 959 note), if we adopt a model of communicated content which requires access to the intentional states of speakers, the kind of access to mental states involved cannot be slow or effortful. We typically hear the meaning of utterances as quickly and directly as we perceive anything in our environment, thus accessing communicative intentions must be fast, automatic, etc. Finally, as noted above, smart behaviour reading is best construed as the result of associative learning about statistical regularities across perceived behaviour, but it is not obvious that this kind of process is one that Kahneman himself would be happy to include as a System 1 process, for he notes (Kahneman 2011: 36) that System 1 is not adept at using purely statistical information, writing Kahneman 2011: 77 that “Statistical thinking derives conclusions about individual cases from properties of categories and ensembles. Unfortunately, System 1 does not have the capability for this kind of reasoning”.

So I want to suggest that while both statistical behaviour reading and abductive mental state attribution have a part to play in action understanding *both* can be fast or slow, effortful or easy, explicit or implicit.^26^ Instead of a System 1/System 2 divide then, what I think the division marks is the different kinds of cognitive processes in play: deductive, computational, formal processes (in statistical learning) versus abductive, inference-to-the-best explanation, informal processes (in mental state attribution). However, while these are very different kinds of thinking, I maintain that it would be a mistake to think either process is necessarily fast or slow, conscious or unconscious, difficult or easy. Though we are much less clear on how to model abductive thinking, we have no reason to expect that this type of paradigmatically human thinking must be slower or more difficult than more constrained and computationally tractable thought.^27^

## Conclusion

Advocates of behaviour reading approaches are right to think that smart behaviour tracking – an appropriate sensitivity to the embodied actions of others – is crucial to human action understanding. However, I’ve argued that the behaviour tracking underpinning action prediction cannot take place in the absence of a background of mental state attribution: to characterise learning situations we need to know how the target views or conceptualises the situation, and to compare a current situation to a learnt one (and thus to apply the right behavioural rule) we need to know something about the mental framework the target brings to the situation. Though we can, it seems, often predict action in light of behavioural contingencies that we may not even be consciously aware of (e.g. predicting that a cyclist will pull out in front of us on the basis of sub-conscious sensitivity to covert behavioural ‘tells’), these physically described phenomena make sense to us only within a richly psychologised framework (one where we assume that the cyclist wants to move off, believes they have room before the next car, etc). This kind of smart behaviour reading is often (though not always) fast, effortless and automatic, it relies on learnt associations between behaviours and outcomes, and it provides one way in which we are able to tailor our actions to our social world. Yet, on closer inspection, I’ve suggested that such behaviour reading in fact relies on a background of mental state attribution, on seeing the other through the lens of classic mental states such as beliefs and desires, as well as cognitive and affective states such as character traits and moods. In practice, using smart behaviour reading to predict actions, far from replacing mental state attribution in the human case, depends upon it. However, I have argued that we should not see this role for mental state attribution as problematic, since we have little reason to think that it is necessarily slower, more effortful or more demanding of conscious control than other forms of understanding.

## Footnotes

1 According to theory-theory accounts typically developed adults are in possession of a theory of mind (something they either acquired, e.g. Gopnik and Meltzoff 1997, or have innately, e.g. Scholl and Leslie 1999). To understand the actions of others we subsume them under the very general psychological laws of our tacit theory, engaging in (usually) subconscious acts of inferential reasoning to predict and explain behaviour. On the opposing, simulation-based view (e.g. Heal 1986; Gordon 1996; Goldman 2006) subjects use their *own* internal planning mechanisms in an imaginative exercise; according to simulation theory understanding others really does involve ‘putting yourself in the other person’s shoes’.

2 I won’t distinguish between embodied views per se and related views which group under the ‘4e’ label, whereby cognition is some combination of embodied, embedded, extended and enactive; see Rowlands 2010 for discussion.

3 This kind of deflationary view of behaviour reading might be thought to fit with an approach like Barsalou’s ‘perceptual symbols theory’ (2003, 2008, 2009), where the neural systems involved in thinking about a given property are the same as the systems involved in perceiving that property.

4 Or again, as Gallagher 2001: 86 writes: “[I]n most intersubjective situations we have a direct, pragmatic understanding of another person’s intentions because their intentions are explicitly expressed in their embodied actions. For the most part this understanding does not require us to postulate some belief or desire that is hidden away in the other person’s mind, since what we might reflectively or abstractly call their belief or desire is directly expressed in their behaviour”.

5 As a reviewer for this journal rightly pointed out, this is a contentious point and will depend on exactly what we take to be required for an embodied approach. For instance, the kind of enactivist model preferred by Hutto and Gallagher, which emphasises the nonrepresentational nature of thought, sits uncomfortably with mirror neuron approaches, which preserve the idea of representation. However other forms of embodied account apparently place less stress on this aspect (e.g. Rowlands 2010) or suggest a reconstrual rather than an outright rejection of representations (e.g. Barsalou’s perceptual symbols theory). On these approaches, what matters to an embodied account is recognising an essential role for the body and motor systems in underpinning thought, of a kind which goes beyond the role accorded to the body in standard Cartesian approaches. On this latter understanding of embodiment, then, it is open to advocates to maintain that the mirror neuron account of action understanding provides a potential neurological basis for embodiment. Connecting embodiment with the mirror neuron system in this way is, I think, fairly common in the literature. So, for instance, the influential Stanford Encyclopaedia entry on embodied cognition lists the discovery of mirror neurons as one of “four evocative examples of phenomena that have motivated embodied cognitive science”, while Caramazza et al. 2014 refer to the mirror neuron theory of action understanding as “one of the most influential examples of embodied cognition theories”.

6 For further discussion of this point, see Borg 2017a.

7 Standard folk psychological models run together explanation and prediction as two sides of the same coin (explanation just being retrospective prediction), however, as Andrews 2012 convincingly argues this need not be the case. We could, and perhaps should, posit different systems for each. Although I won’t pursue this point here, it is worth noting that, although it is less obvious than its role in prediction, a behaviour reading account could perhaps play a role in action explanation (along the lines of the factual explanations of behaviour offered by Perner 1991, Csibra and Gergely 2007, amongst others). So, for instance, consider an explanation of the following form: ‘Why did Karthik get out of the car?’ ‘Because he’s going into the shop’. If ‘going into the shop’ could be read here as a description of behaviour (rather than as a teleological description) this might count as a putative case of explanation via behaviour reading. I’m grateful to Nat Hansen and to an anonymous referee for discussion here.

8 Or again as Povinelli and Vonk 2004: 8–9 put the point: “[I]t must be conceded that the subject’s predictions about the other agent’s future behaviour could be made either on the basis of a single step from knowledge about the contingent relationships between the relevant invariant features of the agent and the agent’s subsequent behaviour, or on the basis of multiple steps from the invariant features, to the mental state, to the predicted behaviour. Without an analytical specification of what additional explanatory work the extra cognitive step is doing in the latter case, there is nothing to implicate the operation of S_b + ms_ over S_b_ alone”.

9 Lurz 2011: 76 puts this step as follows: “Since mental state concepts applied to others are based on observable facts or cues about the other agent’s behaviour or environment, A must apply the mental state concept *m* to B on the grounds of some such fact or cue, about B’s behaviour or environment.” To advertise one conclusion of the paper in advance: I will argue that this premise about the evidential base doesn’t hold in our own case. For human social cognition we have reason to think that the evidential base often needs to make reference to the unobservable mental properties of the target.

10 The thought is that mental state attribution (what Dennett 1971, 1987 terms ‘second-order intentionality’) is a more advanced skill since it requires reasoning about abstract states, like beliefs and desires. The operative principle seems to be Lloyd Morgan’s 1894: 53 canon: “In no case may we interpret an action as the outcome of the exercise of a higher psychical faculty if it can be interpreted as the outcome of the exercise of one which stands lower in the psychological scale”. However, it is worth noting that Lloyd Morgan’s later statement of the principle also makes clear that it would not support a move to a general deflationary view of human social cognition, for he writes 1903: 59: “To this, however, it should be added, lest the range of the principle be misunderstood, that the canon by no means excludes the interpretation of a particular activity in terms of the higher processes, if we already have independent evidence of the occurrence of these higher processes in the animal under observation”. I return to the argument from parsimony in §2.iv.

11 To be clear, the generalising of the Problem to adult human action prediction is not something advocated by Povinelli and colleagues (although it is sometimes hinted at; for instance, Povinelli and Vonk 2006: 7 write that “it seems likely that much human social interaction is supported solely by the features of S_b_ that we have just described”). Rather the extension is suggested here because the form of the argument allows it and because taking the third premise seriously might seem to recommend a wider application: if a lower level (non-mindreading) explanation is possible and indeed is preferable (in terms of parsimony and simplicity) in the nonhuman realm then, the thought is, we would need a positive reason not to apply the same reasoning where we have human action understanding in the absence of explicit evidence of mental state attribution. What this picks up on is the thought (stressed in Halina 2015, see n.24 below) that Povinelli’s Problem has the underlying form of a more general sceptical argument and, as such, may have a wider range than Povinelli himself envisaged.

12 Finally, we *could* also choose to extend the range of the sceptical move embodied in Povinelli’s Problem even further by arguing that verbal behaviour is just one more piece of observable action which falls short of conclusively demonstrating mental state attribution: the fact that a subject *says* something like ‘John believes that there are smarties in the tube’ does not guarantee that that subject has a thought which attributes a mental state to John. However, for the purposes of this paper I’ll put this most radical sceptic to one side (see also Lurz 2011: 30–1 who also appeals to human language use to restrict an extended application of Povinelli’s Problem).

13 Without wishing to go into too much detail here, it might be objected that this point is mistaken since Heyes does consider at least some cases of action prediction tasks. For instance, the last experiment considered in Heyes 2014 concerns preferential looking in a false belief task, precisely the kind of experiment often taken to show tacit perspective taking in action prediction. However, first, I think questions could be raised about the submentalizing explanation offered for this kind of case (which is in terms of participant attention) and, second, we might note that other tests which involve precisely the kind of ‘self-informed belief induction variables’ which Heyes herself recommends as a good way to test for genuine perspective taking (such as Teufel et al. 2010; see n.17 below) do seem to show evidence of implicit mentalizing in these cases.

14 I’m grateful to an anonymous referee for stressing this point.

15 The idea that in such predictions the system is akin to that used for automated systems is also found in Andrews 2012: 51: “I predict the person’s behaviour using the same reasoning as I use for the machine, by making generalizations from past experience and knowing the target’s function”. Though note that Andrews also argues for a greater demotion of belief/desire reasoning than is suggested in this paper (e.g. 2012: Ch.6).

16 Two points to note, however: first, Lurz et al. go on to note with apparent approval (in their n.7) suggestions by Apperly and Butterfill that infant success on false belief tasks can be explained by their possession of simple behavioural rules. However this suggestion apparently runs counter to Lurz et al.’s earlier claim (2014: 442) that success on false belief tasks can’t be captured by simple behavioural rules (as it is this which, for them, is supposed to explain the fact that primates fail false belief tasks). Second, although I agree with the overall point of the quote from Lurz et al. above, it is easy to read the quote as also implying something about the human mental state attribution system – namely that it (unlike a behaviour reading system) is slow and effortful. I want to deny any claim of this kind. Rather the suggestion will be that we have two systems: a behaviour reading one and a mindreading one, and both systems are capable of acting in a quick, effortless and automatic way, or in a slow, deliberative, conscious way. See §3 below.

17 Thus the fact that the same objection surfaces again here might make us re-evaluate the earlier claim that behaviour-tracking models can avoid being classed as Behaviourist accounts.

18 In this respect, see Meltzoff’s 1995 experiments with copying behaviour in children. In these experiments children were exposed to novel toys and, in the crucial contexts, witnessed an experimenter apparently trying but failing to perform a given action with the toy (e.g. presented with a toy containing a hook and a loop, the experimenter mimed trying to put the loop over the hook, but in fact dropping the loop ineffectually to one side of the toy). Children then given the toy performed not the behaviour they had actually witnessed (e.g. picking up and dropping the loop) but the action the experimenter seemed to have been aiming at, in this case hanging the loop successfully on the hook. Meltzoff’s suggestion is that children are able to ‘see through the action to the intention’, highlighting the idea that what we get from witnessing the behaviour of others – what learnt associations we acquire – often depends on how we characterise the mental states of the performer.

19 That the way in which a subject conceptualises the mental states of others can affect even the most automatic-seeming of behavioural responses may also be shown by recent experimental work. For instance (as discussed in Jacob 2018 forthcoming), Teufel et al. 2010 exposed one set of subjects to a pair of goggles with transparent lenses and another set of subjects to an outwardly indistinguishable pair with opaque lenses. Both sets of subjects then watched an experimenter (face on) don one of the pairs of goggles and look around. Those in the former group (exposed to transparent goggles only) had their gaze cued by the head movements of the experimenter, but those in the second group (exposed to opaque goggles) did not, apparently showing that gaze direction cued by the head movements of a conspecific depend on a background assumption that the conspecific can see.

20 In support of this, we might note that experimental work shows that where there are small-scale identifiable kinematic differences between actions, subjects are nevertheless unable to predict ensuing actions on the basis of these differences alone. So for instance, in work looking at reach-to-eat versus reach-to-place, Naish et al. 2013 found that subjects witnessing just the grasp portion of a movement which was in fact either a grasp-to-eat or a grasp-to-place were unable to predict correctly what would happen next (i.e. identify the act as the initial stage of a grasp-to-eat, say), even though subtle kinematic differences between the kinds of gestures were found.

21 This point connects to Spaulding’s 2014 objection that behaviour reading models cannot cope with counterfactual thinking about what someone might have done in a different situation; see also de Bruin and Kästner 2012.

22 Or again, I might consider Jill to be honest even if I know she has lied on the three previous occasions when she’s made an assertion (for instance, if these three assertions were made to axe-wielding maniacs inquiring about someone’s location). The point again is that individuating relevant situations for trait-attribution seems to require a mentalized perspective on situations.

23 For a discussion of the extent of reasoning about speaker intentions in communication see Geurts and Rubio-Fernández 2015, Borg 2016, 2017b.

24 For a further challenge to the theoretical/ methodological assumptions behind Povinelli’s Problem, see Halina 2015. Halina objects to Povinelli’s Problem primarily on the grounds that it is a version of a general sceptical problem known as ‘the theoretician’s dilemma’ and, as such, it imposes an evidential requirement on claims concerning animal mindreading that advocates should not be required to meet. I agree with Halina’s diagnosis (and I’m grateful to a reviewer for this journal for pointing me to the paper, which I wasn’t previously aware of) and take the objections raised here to behaviour reading accounts of human action understanding to be complimentary to her objections to behaviour reading accounts of non-human action understanding (indeed I think it is this kind of recognition of the underlying sceptical assumptions of the Problem which might underlie the extension of the range of the Problem from the non-human animal realm, through infants to adult behaviour, as suggested in §1.ii).

25 For instance, this seems to motivate Fiebich and Coltheart 2015, where they aim to introduce a “pluralistic two-system account of social understanding that advocates the view that individuals expect, predict or explain the behaviour of other people in everyday life in various ways” (2015: 236) and that these ways reflect two different systems of cognitive processing, in the sense of Kahneman 2011. So behaviour tracking is a fast, automatic, heuristic driven process typical of System 1, while belief-desire reasoning is a slow, deliberative System 2 process. Similarly Apperly and Butterfill 2009 posit a System 1 process which trades in psychological-like states, such as ‘encountering’ and ‘registering’, which don’t require consideration of mental states as such (e.g. don’t require full-blown belief-desire attribution), and which can thus meet the requirements of speed and automaticity.

26 This is to agree with Carruthers 2013, though he maintains that humans need not be held to utilise a behaviour reading system in addition to a mindreading system

27 Thus the model I want to advocate here is an extension of the variety of two-system model of cognition I’ve advocated elsewhere, see Borg 2004, 2012. The claim that mental state attribution could be fast, automatic, etc., is rejected by Apperly and Butterfill 2009, however their argument for this claim is not absolutely transparent (for much fuller discussion, see Jacob 2018 forthcoming).
